# Life events as predictors for disability pension due to musculoskeletal diagnoses: a cohort study of Finnish twins

**DOI:** 10.1007/s00420-019-01505-5

**Published:** 2019-12-11

**Authors:** Sanna Kärkkäinen, Karri Silventoinen, Pia Svedberg, Annina Ropponen

**Affiliations:** 1 Institute of Public Health and Clinical Nutrition, Faculty of Health Sciences, University of Eastern Finland, Kuopio, Finland; 2 Department of Social Research, University of Helsinki, Helsinki, Finland; 3 Division of Insurance Medicine, Department of Clinical Neuroscience, Karolinska Institutet, Stockholm, Sweden; 4 Finnish Institute of Occupational Health, Helsinki, Finland

**Keywords:** Life change events, Musculoskeletal disorders, Disability pension, Twins

## Abstract

**Purpose:**

Musculoskeletal diagnoses (MSD) are one of the largest diagnostic groups for disability pensions (DP). This study investigated the associations between life events and DP due to MSD, considering sociodemographic, health, and familial factors.

**Methods:**

The study sample included 18,530 Finnish twins, 24–64 years old at baseline, who responded to a questionnaire in 1981 including a 21-item life event inventory. Information on DP with diagnosis codes (ICD codes: M00–M99) were obtained from the official national pension registers. Life events were divided into family- and work-related events. “Positive change in life” was analyzed separately. Cox proportional hazards models were used to calculate hazard ratios (HR) with 95% confidence intervals (CI).

**Results:**

During the follow-up of 23 years, 1273 (7%) individuals were granted DP due to MSD. In discordant pair analysis, family-related events (≥ 4 events) increased (HR 1.63, 95% CI 1.31, 2.03) and the absence of such events decreased (HR 0.68, 95% CI 0.48, 0.95) the risk of DP due to MSD. For work-related events (≥ 3 events), the risk estimates were non-significant when controlling for familial factors. Having had a positive change in life decreased the risk of DP due to MSD (HR 0.79, 95% CI 0.65, 0.96) while controlling for familial confounding, but were non-significant in the full model controlling for various covariates (HR 0.91, 95% CI 0.75, 1.12).

**Conclusions:**

The associations between life events and the risk of DP due to MSD are complex and potentially affected by familial and other confounding factors including sociodemographics and health.

## Introduction

In Finland, as in the OECD countries in general, musculoskeletal diagnoses (MSD) are the second largest diagnosis group after mental health diagnoses for disability pensions (DP) causing early exit from the labor market with negative economic consequences for both the individual and the society as a whole (OECD 2010). Life events include both neutral and major stressful events, such as changing residence, interpersonal conflicts at work, or the death of a close one (Holmes and Rahe 1967). In addition, both positive and negative life events may be considered as additional stressors beyond normal everyday life that may lead to increased vulnerability to illness (Lazarus 1984), being linked with suboptimal health, but subsequently also with consequences of chronic illnesses such as DP (Appelberg et al. 1996; Cleland et al. 2016; Zhang et al. 2017). At present, although some indications exist that life events may affect future health (Appelberg et al. 1996; Kivimäki et al. 2002; Lin et al. 2013), knowledge on the association between life events and DP due to MSD is limited.

Earlier studies on the risk of DP have included information on the effect of both separate life events and further combined life events. For example, in a study among municipal workers, interpersonal conflicts at work increased the risk of DP among women, especially among those with simultaneous marital conflicts (Appelberg et al. 1996). Periods of unemployment have been found to increase the risk of DP in general (OECD 2010), and stressful life events, categorized as the death of a close one, experienced violence, and financial problems, were shown to increase the risk of sickness absence among men (Kivimäki et al. 2002). Since sickness absence is a strong predictor of DP, and DP due to MSD is one of the main diagnosis groups for DP (OECD 2010), these findings reflect potential interplay between life events and the risk of DP due to MSD. Additionally, for DP due to MSD, psychosocial environment at workplace, measured as organizational justice, has shown association with DP due to MSD (Juvani et al. 2016). Moreover, evidence exist that sociodemographic and health-related risk factors are associated with both stressful life events and the risk of DP due to MSD. For example, having gone through divorce or experienced the death of a spouse have been shown to increase smoking and decrease physical activity (Osler et al. 2008; Engberg et al. 2012). Additionally, lower socioeconomic status and education level (Bruusgaard et al. 2010; Ropponen et al. 2011), negative health behaviors, the presence of other diseases, musculoskeletal pain (Ropponen et al. 2011, 2013) and being obese or overweight (Ropponen et al. 2016) have been shown to predict DP due to MSD. Furthermore, among the negative life events, periods of unemployment increase the risk of DP due to MSD, but this association is shown to be confounded by sociodemographic factors (Kärkkäinen et al. 2013). Hence, the pathway from the life events to DP due to MSD might be linked with the underlying disease for DP grant or with various other factors including sociodemographic or health-related factors.

Moreover, in previous Nordic studies, genetic factors have been found to account for 35–37% of variance in DP due to MSD (Narusyte et al. 2011; Harkonmäki et al. 2008), whereas regarding life events, the role of genetics has been found to be 27% (Kendler and Baker 2007). This refers to the potential presence of familial confounding. A twin study setting allows controlling for this possible influence of familial confounding, including shared genetic factors and unmeasurable shared environmental factors, mainly childhood family environment, of twin siblings.

In this study, as a theoretical framework, all the queried life events, regardless of being generally considered negative or positive, were assumed indicators for overall stress as they all present environmental change beyond normal everyday life (Holmes and Rahe 1967; Lazarus 1984; Folkman 2008). These events, especially in the case of accumulation of events, may lead to increased vulnerability to illness (Lazarus 1984). The potential associations between stressful life events and DP due to MSD is considered to potentially differ for family- and work-related life events as these can be considered as different causes of stress. It was hypothesized, that work and family events may have different impact on health due to different personal significances when at work or in a family setting (Reitzes and Mutran 2002) together with coping abilities (Hur et al. 2012; Park and Iacocca 2014). Hence, an assumption was made that the accumulations of life events, even at one point in time, may predispose an individual to future health risks due to perceived stress and coping abilities, where social roles are potentially also significant. Consequently, family- and work-related events may differ in their associations with DP due to MSD.

The aim of this study was to investigate the associations between life events, separately for family- and work-related events, and DP due to MSD, in a cohort of 18,530 twin individuals with 23 years of follow-up data. Our specific aim was to analyze these associations while controlling for sociodemographic and health-related covariates, and for familial confounding.

## Methods

The study population was derived from the Finnish Twin cohort including same-sex Finnish twin pairs born before year 1958 (Kaprio and Koskenvuo 2002). Comprehensive baseline questionnaires were sent to all twins in the cohort in 1975 and 1981 (response rates 89% and 84%, respectively). Follow-up time was from January 1st 1982 to December 31st 2004. Questionnaire information from 1981 was used in all analyzes when available. Socioeconomic status and education were available from 1975. The original sample included 13,888 twin pairs. For this study, those who were alive and had not retired or emigrated before the start of follow-up, and who had responded to the 1981 questionnaire, were included. The final study sample included 18,530 twins (51% women), comprised of 2498 (56% women) monozygotic (MZ) and 5127 (52% women) dizygotic (DZ) same-sex twin pairs. Zygosity was determined based on questions of similarity of physical appearance in childhood, which has been shown to be highly accurate in distinguishing between MZ and DZ twins (Sarna et al. 1978).

### Assessment of life events

The questions on life events were based on a 21-item life event inventory derived from the Holmes and Rahe (1967) stressful life event scale, with adjustments to consider typical national life events (Lillberg et al. 2003). Life events were further divided into family- and work-related events based on Pearson correlation coefficients between each separate life events in the study cohort (Appendix A). The single life event “positive change in life” was analyzed separately as it had similar associations with both family- and work-related events. Another single event, “illness or injury causing over 3 weeks of work disability” was analyzed separately, since it may directly influence the risk of DP due to MSD (Finnish Centre for Pensions 2016).

In the questionnaire, the response options were “not at all”, “during the last 6 months”, “during the last 5 years”, and “more than 5 years ago” for each life event. For this study, the events were trichotomized as “no event”, “event within 5 years” and “event more than 5 years ago”.

The life events were categorized based on the frequencies of the events in the study cohort. The family-related events (0–14 events) were categorized to “no events”, “1–3 events” or “4 or more events”. Work-related events (0–5 events) were categorized to “no events”, “1–2 events” or “3 or more events”. Additionally, a summary variable “all events” (0–20 events) was created and was categorized as “no events”, “1–3 events” and “4 or more events”. The reference classes were “1–3 events” for family-related events and all events and “1–2 events” for work-related events. Furthermore, for family-related, work-related, and all events, also a dichotomic categorization “no events within 5 years” or “any event within 5 years” was created. All reported events within 5 years were included instead of limiting to only those who had answered all questions in the life event inventory.

### Assessment of covariates

#### Sociodemographic factors

Socioeconomic status included six categories: upper and lower non-manual workers, skilled and unskilled manual workers, farmers and others including students, conscripts, and those otherwise not classified (Statistics Finland 1974). Education level was measured as years of education based on the highest attained educational level and used as a continuous variable. Marital status was dichotomized as married (those living with a partner, including cohabitating, married or re-married) or single (those living alone, including unmarried, divorced and widowed).

#### Health-related factors

Body mass index (BMI) was calculated from self-reported height and weight (kg/m^2^) and used as a continuous variable. The reliability of self-reported BMI has been found to be good in this cohort (Korkeila et al. 1998). Self-reported smoking status was categorized as “never smoked”, “occasional smoker”, “ex-smoker” or “current smoker”. Leisure-time physical activity was an assessment of overall physical activity level, where monthly frequency, mean duration, and mean intensity of physical activity were computed into metabolic equivalent (MET) values (Kujala et al. 1998). Musculoskeletal pain was assessed with a question on pain in either the neck, shoulder or low back area that had impaired work ability in recent years. Response choices “yes” or “no” for each location were dichotomized as having no pain locations or having at least one. For the frequency analgesic use, analgesic use during the last year was queried with response options of “less than 10 days” or “10 or more days”. For the presence of other diseases, participants answered “yes” or “no” to having a specific chronic disease and were categorized as having no chronic disease or having at least one. The chronic diseases queried included bronchitis, pulmonary emphysema, asthma, allergic rhinitis, allergic dermatitis, urticaria, hypertension, angina pectoris/myocardial infarct, stroke, gastric ulcer, gallstones, diabetes and gout. Alcohol consumption was measured with a quantity–frequency questionnaire of beverage-specific use and grouped into four classes: abstainers, light users, moderate users and heavy users following the sex-specific criteria of the National Institute on Alcohol Abuse and Alcoholism (Järvenpää et al. 2005; Romanov et al. 1987).

### Missing information

Before creating the summary variables for life events, individual questions were analyzed for missing information. In total, there was 4–9% missing information in the individual questions of life events in the whole study cohort. Those who were older had more missing information and women had slightly more missing information than men. For zygosity, there was no difference in missingness. When computing the summary variables, the option “event more than 5 years ago” was coded separately as an own category to avoid unnecessary loss of data. For these summary variables, missing was ≤ 4%. From covariates, pain locations had 27%, socioeconomic status 9% and leisure time physical activity 8% of missing data. In other covariates the missing was very low, ≤ 5%. Due to missing data, the models controlling for various covariates included 550–599 individuals.

### Follow-up data

Registry-based information on DP with diagnostic codes (International Classification of Diseases, ICD 8/9 and 10th revisions) during the follow-up period from 1982 to 2004 was obtained from the Social Insurance Institution and the Finnish Centre for Pensions. DP can be granted to a person with long-term work impairment, requiring at least 1 year of sickness absence from work due to a medically confirmed diagnosis. During the follow-up time, from 1986 until gradually abolished by year 2005, also early retirement pension was available for employees 58–64 years of age, which required medically confirmed illness, disease or injury, that crucially restricted work capacity. No major changes for disability pension criteria were introduced during the follow-up time of this study (Finnish Centre for Pensions 2016). All diagnoses were transformed into ICD-10 rubrics M00–M99. Information on dates of emigration and death was obtained from regular updates from the Population Register of Finland. The unique personal identification codes of all Finnish citizens were used for the record linkage.

### Statistical analyzes

To analyze the associations between life events and DP due to MSD, Cox proportional hazards models computing hazard ratios (HR) with 95% confidence intervals (CI) were used. The analysis of the whole cohort, with twins treated as singletons, was performed by clustering with twin pair identity to adjust for standard errors to take into account within-pair correlations (Williams 2000). All analyzes were adjusted for age at baseline. To minimize the potential effect of having both DZ and MZ twins in the models, zygosity was also adjusted for. Results were analyzed first for men and women separately. As there were no significant sex differences (*p* < 0.05), both sexes were pooled together. Stratification for sex was used to allow for separate baseline hazards for men and women (Messing et al. 2009). The proportional hazards assumption for individual variables was tested with the graphical observation of “log–log” curves for categorical variables. No violation of the assumption was detected.

Several potential confounders were considered for their possible effect on the association between life events and DP due to MSD. With backward selection, only factors with statistically significant associations at *p* < 0.05 with DP due to MSD were included in the final full model. The full model was conducted separately for family- and work-related events and for single life events “positive change in life” and “illness or injury causing over 3 weeks of work disability”.

After these individual level analyses, discordant twin pair analyzes were conducted to analyze whether familial factors (genetics and shared environment) have an effect on the found associations. Only complete twin pairs, from whom one twin had been granted DP due to MSD during follow-up and other had no DP (i.e., discordant twin pairs), were included. Conditional Cox proportional hazards models were used with stratification by twin pair to allow each pair to have an own baseline hazard. Discordant twin pair analyzes were also conducted separately for MZ and DZ twins.

When interpreting the influence of familial confounding, the models where twins were treated as singletons were used as the basis of comparison. If the risk estimates attenuate in the within-pair analyses including discordant twin pairs, it would indicate familial confounding (McGue et al. 2010). Furthermore, in analyzes conducted separately for DZ and MZ pairs, the difference in risk estimates would indicate genetic influence: the effect of shared family environment is similar for DZ and MZ pairs, but they differ in their genetic match, since DZ twins share 50% of their segregating genes and MZ twins 100%.

Additional analyzes were also conducted (data not shown): For both family- and work-related events, events were divided based on the Holmes and Rahe scale (Holmes and Rahe 1967) into “no events”, “most stressful events” and “stressful events” (Appendix A). Additionally, for work-related events “interpersonal conflict at work” and “loss of a job” were combined as their own category to analyze the influence of negative work-related events against other work-related life events. In these additional tests, no significant associations were found for work-related major and minor stressful events, or negative and positive events. Instead for family-related events, the major life events were in line with the results for having any family-related events with statistically significant results shown only for discordant twin pairs (Tables 3, 4), while for minor life events, all results were non-significant (data not shown).

The effect of the reference group was tested by comparing results excluding or including “those with DP due to other reason than MSD”. Since this did not influence the risk estimates, both those without any DP or DP due to other reason than MSD were combined as reference group to maximize the sample size (data not shown). In addition, no significant interactions with sex and family- or work-related life events were found. The baseline descriptive statistics for background, covariates and life events in the Tables 1, 2 are presented for those without any DP (no DP) and for those with DP due to MSD.

**Table 1:** Baseline means with standard deviations (SD) and percentages (%) for those men and women granted disability pension (DP) due to musculoskeletal diagnoses (MSD) and those without any DP during follow-up between 1982 and 2004

	DP due to MSD (*n* = 1273)		no DP (*n* = 15,327)	
	Men (*n* = 632)	Women (*n* = 641)	Men (*n* = 7306)	Women (*n* = 8021)
	Mean (SD)	Mean (SD)	Mean (SD)	Mean (SD)
Age at baseline 1 Jan 1982 (range 24–64 years)	43.3 (8.8)	44.0 (8.3)	36.1 (9.8)	36.2 (10.4)
Follow-up time (years)	10.2 (5.7)	10.7 (6.1)	19.3 (6.4)	19.4 (6.4)
Education in 1975 (years)	6.5 (1.3)	6.7 (1.5)	8.0 (2.8)	8.2 (2.8)
Body Mass Index, BMI [kg/m^2^]	25.7 (3.0)	24.8 (3.9)	24.2 (2.9)	22.6 (3.3)
Leisure-time physical activity (metabolic equivalent, MET)	4.4 (4.3)	4.1 (3.5)	4.6 (4.4)	4.1 (3.5)
	%	%	%	%
Socioeconomic status in 1975				
Upper non-manual worker	3	1	9	6
Lower non-manual worker	17	23	18	18
Skilled manual worker	46	36	37	42
Unskilled manual worker	11	17	8	10
Farmer	8	14	7	7
Others	0	3	8	1
Marital status				
Married	80	74	72	71
Presence of other diseases				
Any other chronic disease	63	67	42	51
Any musculoskeletal pain				
Yes	59	59	34	35
Use of analgesics				
< 10 days per year	62	50	80	68
≥ 10 days per year	30	41	17	27
Smoking status				
Never smoked	23	65	33	58
Occasional smoker	5	2	4	2
Former smoker	31	12	26	17
Current smoker	42	20	36	22
Alcohol consumption				
Abstainer	8	30	8	21
Light	5	15	4	14
Moderate	39	32	41	36
Heavy	41	17	42	25

**Table 2:** Baseline percentages of life events within 5 years of baseline questionnaire for those men and women granted disability pension (DP) due to musculoskeletal diagnoses (MSD), and those without any DP during follow-up, between 1982 and 2004

	DP due to MSD (*n* = 1273)		No DP (*n* = 15,327)	
	Men (*n* = 632)	Women (*n* = 641)	Men (*n* = 7306)	Women (*n* = 8021)
	%	%	%	%
Family-related events				
0 events	6	4	5	5
1–3 events	67	64	62	60
≥ 4 events	19	20	26	28
Work-related events				
0 events	35	37	32	36
1–2 events	44	37	44	41
≥ 3 events	6	8	14	12
Other events				
Positive change in life	18	22	32	36
Illness or injury causing over 3 weeks of work disability	33	27	16	14
All events^a^				
No events	5	3	3	3
1–3 events	51	49	40	40
≥ 4 events	38	38	52	53

^a^Does not include event “illness or injury causing over 3 weeks of work disability”

All the analyzes were performed with Stata statistical software, version 13.1, Stata Corp LLC, College Station, TX, USA.

## Results

During the 23 years of follow-up, 7% (1273 individuals, 50% women) of the total study sample of 18,530 (51% women) participants were granted DP due to MSD. Those granted DP due to MSD were, on average, 43.7 years old [standard deviation (SD) 8.6] at baseline, with a mean follow-up time of 10.5 (SD 5.9) years and had, on average, 6.6 (SD 1.4) years of education, making them older with shorter follow-up times and having less education than those without any DP (Table 1). Regarding family- and work-related life events, the number of individuals having many events was lower among those with DP due to MSD compared to those with no DP (Table 2). Main categories within DP due to MSD were low back diagnoses (M40–M54; 31%) and osteoarthritis (M05–M19; 34%).

In the analyses of the whole cohort, family-related life events showed no significant associations with DP due to MSD (Table 3). For work-related life events, a decreased risk for DP due to MSD was seen among those with many work-related events in the age and sex adjusted model (HR 0.75, 95% CI 0.60–0.94). However, when adjusted for covariates, this association diluted to statistical non-significance (HR 0.75, 95% CI 0.54–1.04). The absence of family- or work-related events had no significant associations with DP due to MSD.

**Table 3:** Hazard ratios (HR) and 95% confidence intervals (CI) of the association between life events within 5 years of baseline questionnaire and disability pension (DP) due to musculoskeletal diagnoses (MSD)

	DP due to MSD			
	Adjusted for age, sex and zygosity (*n* = 1273)		Full model^a^ (*n* = 599)	
	HR	95% CI	HR	95% CI
Family-related events				
No events	0.91	0.71, 1.17	1.04	0.73, 1.50
1–3 events	1		1	
≥ 4 events	1.01	0.87, 1.16	1.10	0.91, 1.28
Any event within 5 years vs no events	1.10	0.85, 1.41	0.98	0.69, 1.40
Work-related events				
No events	0.92	0.81, 1.05	0.96	0.80, 1.15
1–2 events	1		1	
≥ 3 events	**0.75**	**0.60**, **0.94**	0.75	0.54, 1.04
Any event within 5 years vs no events	1.03	0.91, 1.17	1.00	0.84, 1.19
Other events				
Positive change in life	**0.79**	**0.68**, **0.91**	0.91	0.75, 1.12
Illness or injury causing over 3 weeks of work disability	**2.25**	**1.99**, **2.56**	**1.73**	**1.44**, **2.08**
All events^b^				
No events	0.93	0.70, 1.24	0.96	0.63, 1.45
1–3 events	1		1	
≥ 4 events	0.90	0.80, 1.01	0.91	0.76, 1.08
Any event within 5 years vs no events	1.02	0.77, 1.36	1.01	0.67, 1.53

Statistically significant results are in bold

^a^Adjusted for age, zygosity, socioeconomic status, years of education, body mass index (BMI), smoking, presence of any chronic disease, use of analgesics, any musculoskeletal pain. Number of individuals in a final model varied between 550 and 599 depending on the missing data in covariates

^**b**^Does not include event “illness or injury causing over 3 weeks of work disability”

In the analyses of discordant twin pairs controlling for familial confounding, having many family-related events increased the risk (HR 1.72, 95% CI 1.33–2.23) (Table 4). For the absence of events, a decreased risk for DP due to MSD was seen (HR 0.68, 95% CI 0.48–0.95). For many work-related life events (HR 1.12, 95% CI 0.83–1.51) and for the absence of work-related events (HR 0.97, 95% CI 0.81–1.16), no significant associations were found.

**Table 4:** Hazard ratios (HR) and 95% confidence intervals (CI) of the association between life events within 5 years of baseline questionnaire and disability pension (DP) due to musculoskeletal diagnoses (MSD) during follow-up in discordant twin pairs, separately for monozygotic (MZ) and dizygotic (DZ) twin pairs

	DP due to MSD					
	All discordant pairs^a^ (*n* = 787 pairs)		MZ (*n* = 201 pairs)		DZ (*n* = 586 pairs)	
	HR	95% CI	HR	95% CI	HR	95% CI
Family-related events						
No events	**0.68**	**0.48**, **0.95**	**0.48**	**0.25**, **0.94**	0.79	0.53, 1.17
1–3 events	1		1		1	
≥ 4 events	**1.63**	**1.31**, **2.03**	1.37	0.89, 2.12	**1.72**	**1.33**, **2.23**
Any event within 5 years vs no events	**1.59**	**1.14**, **2.22**	**2.11**	**1.08**, **4.10**	1.41	0.96, 2.09
Work-related events						
No events	0.97	0.81, 1.16	0.95	0.66, 1.37	1.00	0.81, 1.23
1–2 events	1		1		1	
≥ 3 events	1.12	0.83, 1.51	0.93	0.56, 1.56	1.23	0.85, 1.78
Any event within 5 years vs no events	1.05	0.88, 1.25	1.04	0.73, 1.49	1.02	0.84, 1.25
Other events						
Positive change in life	**0.79**	**0.65**, **0.96**	0.89	0.59, 1.33	**0.76**	**0.61**, **0.94**
Illness or injury causing over 3 weeks of work disability	**1.96**	**1.60**, **2.40**	**1.92**	**1.33**, **2.76**	**1.96**	**1.54**, **2.49**
All events^b^						
No events	0.92	0.63, 1.34	0.50	0.22, 1.12	1.17	0.75, 1.84
1–3 events	1		1		1	
≥ 4 events	1.14	0.95, 1.36	0.91	0.63, 1.31	**1.23**	**1.00**, **1.51**
Any event within 5 years vs no events	1.13	0.78, 1.65	1.98	0.88, 4.42	0.92	0.59, 1.44

Statistically significant results are in bold

^a^One twin in a pair has been granted DP due to MSD, the other twin has no DP due to MSD

^b^Does not include event “illness or injury causing over 3 weeks of work disability”

The life event, “positive change in life”, showed decreased risk for DP due to MSD in the age and sex adjusted model (HR 0.79, 95% CI 0.68–0.91), but was statistically non-significant in the model controlling for all covariates (HR 0.91, 95% CI 0.75–1.12) (Table 3). In the discordant twin pair analysis, risk estimates were similar to those from the analysis of the whole cohort. For the life event “illness or injury causing over 3 weeks of work disability”, an increased risk of DP due to MSD was seen in the model adjusting for covariates (HR 1.73, 95% CI 1.44, 2.08) and for familial confounding (HR 1.96, 95% CI 1.60, 2.40). When all life events were combined, the associations were non-significant (Tables 3, 4). The statistical models explained the variation of outcomes (R2) for 8% in the model adjusted for sex, age and zygosity, and 43% in the final full model adjusted for all covariates.

## Discussion

This study investigated the associations between family- and work-related life events and the risk of DP due to MSD in a cohort of 18,530 twins with 23 years of follow-up. Specifically, we investigated the associations while controlling for various covariates and for familial confounding. Two single life events, “positive change in life”, and “illness or injury causing over 3 weeks of work disability” were analyzed separately. We found no independent association between family- or work-related life events and DP due to MSD in contrast to previous findings (Appelberg et al. 1996; Bergh et al. 2007; Kivimäki et al. 2002). However, our results were in line with studies suggesting that familial confounding may affect the associations between life events and DP due to MSD (Harkonmäki et al. 2008; Narusyte et al. 2011; Kendler and Baker 2007). Additionally, although several confounding factors including sociodemographic and health may play a role too, previous research support our finding that life event “positive change in life” may decrease the risk of DP due to MSD (Peled et al. 2008; Heikkinen et al. 2017).

In the analysis of the whole cohort, no significant associations were found regarding family-related life events. Another main factor of interest, work-related life events, showed a trend that having many events were in the direction of being protective for the risk of DP due to MSD although this was not statistically significant when covariates were controlled for. The absence of family- or work-related events were not significantly associated with DP due to MSD. In earlier studies, stressful work-related events have been shown to be associated with poorer general health independently of being a manual or non-manual worker among male industry workers (Rose et al. 2006). Also contrary to our findings, a period of unemployment, which can be considered as a stressful life event, has been shown to increase risk of DP (OECD 2010).

However, while accounting for familial confounding (i.e., genetics and shared environment, mainly in childhood), several family-related life events were associated with increased risk of DP due to MSD, whereas the absence of family-events decreased the risk. This may potentially reflect familial influence in the tendency of having many events (Kendler 2001; Hammen 2016). Work-related life events had no significant associations with DP due to MSD when familial confounding was considered. Previous research has shown moderate genetic influences both in the risk of DP due to MSD (Harkonmäki et al. 2008; Narusyte et al. 2011) and in the frequency of life events (Kendler and Baker 2007).

The effect of a single life event, “positive change in life”, that showed similar correlations with family- and work-events, was found to be associated with lower risk of DP due to MSD, and uninfluenced by familial confounding. The association diluted to non-significance when sociodemographic and health related covariates were controlled for. This dilution may reflect that life circumstances and other changes in life may play a role. For example, in previous studies, happiness and positive life events have been shown to decrease risk of breast cancer (Peled et al. 2008) and breast cancer mortality (Heikkinen et al. 2017). Although cancer is a very different disease than chronic musculoskeletal disorder, these findings together suggest that perceived positive change in life may be an indicator of a factor or factors of beneficial for future health. Another single life event analyzed separately, “illness or injury causing over 3 weeks of work disability” was found to be a direct (i.e., independent from various covariates, including familial ones) risk factor for DP due to MSD. As long-term sickness absence is a general requirement for being eligible for DP, this finding was according to expectations (Finnish Centre for Pensions 2016).

This study was based on to the consideration that family- and work-related events may differ in their associations with DP due to MSD (Reitzes and Mutran 2002; Hur et al. 2012; Park and Iacocca 2014). The division to family- and work-related events was conducted through the analyses within the cohort. We analyzed also all life events together but found no association with DP due to MSD. Hence, it seems that family events are associated with the risk of DP due to MSD but not the total frequency of events. Other considerations for our findings include that family-related life events may reflect more permanent changes in one’s life than work-related life events, or the difference between acute and chronic stressors (Hammen 2016). More knowledge is needed on the general patterns behind accumulation of family-related life events, to meaningfully account for such in a population level, in efforts to decrease risk of early exit from working life due to DP due to MSD.

The strengths of this study include that we had a large twin cohort with registry information on DP with 23 years of follow-up and, hence, no loss to follow-up. The twin cohort is representative of the Finnish adult population (Kaprio and Koskenvuo 2002). Furthermore, we were able to take into account several sociodemographic and health-related factors from our comprehensive questionnaire information. Moreover, the influence of life events to different health outcomes have been relatively widely studied but information on how life events are associated with DP due to MSD is scarce. Further, using the twin design allowed us optimally to take into account familial factors (both genetic factors and shared environment mainly in the childhood). In addition, due to age of individuals in our sample, only adulthood life events were included, capturing the same life period among all participants. Limitations of the study include that we were not able to control for possible recall bias and, in some categories, the number of cases was low. Also, we had relatively large amount of missing data in some of the covariates. Additionally, as our study included individuals aged 23–64 years, and events that had occurred within the last 5 years were queried, this wide window may have diluted some effects. Moreover, the division of life events to family- and work-related events was based on information received from the twin cohort, and hence, is limited to this cohort. Further limitations include that we had information only from one point at time and no information on potential changes on health behavior or development of overall accumulation of life events during follow-up. Overall, our findings that both family and work-related events in their associations to DP due to MSD were influenced by familial confounding add to the current knowledge and suggest that familial confounding should be considered in this type of studies investigating risk factors for DP. We consider our findings to more likely be an under- rather than overestimation. In regards of generalizability, our results might be most applicable to other Nordic countries with similar social welfare systems, and less in other countries.

## Conclusions

Family- and work-related life events seem to be complex in their associations with DP due to MSD and are potentially affected by familial confounding. The event “positive change in life” seems to play a role for the risk of DP due to MSD, although the effect of sociodemographic factors and health should be noted.

## Appendix A Life events divided into family- and work-related events

**Table:**

Family events (14 questions)
**Death of spouse**
**Death of friend**
**Change in health of family member**
**Sexual difficulties**
**Financial problems**
**Gain of new family member**
**Divorce or separation**
Interrupted pregnancy in family
Change in residence
Family member leaving home
Serious conflict in close relationship
Change in number of arguments with spouse
Taking a loan (more than half of yearly income)
Living away from spouse due to work
Work events (5 questions)
**Loss of a job**
**Change to different kind of work**
Interpersonal conflict at work
Increase in responsibilities at work
Increase in amount of work
Both (1 question)
Positive change in life
Other
Illness/injury that caused over 3 weeks of work disability

Response options to above questions were: “not at all”, “during last 6 months”, “during last 5 years”, “more than 5 years ago”. Events that had occurred during the last 5 years (including the last 6 months) were included in the analysis. Those events that are most stressful, according to the Holmes and Rahe (1967) life event scale, are in bold
